# Protective Effects of Paricalcitol on Peritoneal Remodeling during Peritoneal Dialysis

**DOI:** 10.1155/2015/468574

**Published:** 2015-10-29

**Authors:** Andrea W. D. Stavenuiter, Karima Farhat, Marc Vila Cuenca, Margot N. Schilte, Eelco D. Keuning, Nanne J. Paauw, Pieter M. ter Wee, Robert H. J. Beelen, Marc G. Vervloet

**Affiliations:** ^1^Department of Molecular Cell Biology & Immunology, VU University Medical Center, 1081 BT Amsterdam, Netherlands; ^2^Department of Nephrology, VU University Medical Center, 1007 MB Amsterdam, Netherlands; ^3^Institute for Cardiovascular Research VU (ICaR-VU), VU University Medical Center, 1007 MB Amsterdam, Netherlands

## Abstract

Peritoneal dialysis (PD) is associated with structural and functional alterations of the peritoneal membrane, consisting of fibrosis, angiogenesis, and loss of ultrafiltration capacity. Vitamin D receptor activation (VDRA) plays an important role in mineral metabolism and inflammation, but also antiangiogenic and antifibrotic properties have been reported. Therefore, the effects of active vitamin D treatment on peritoneal function and remodeling were investigated. Rats were either kept naïve to PDF exposure or daily exposed to 10 mL PDF and were treated for five or seven weeks with oral paricalcitol or vehicle control. Non-PDF-exposed rats showed no peritoneal changes upon paricalcitol treatment. Paricalcitol reduced endogenous calcitriol but did not affect mineral homeostasis. However, upon PDF exposure, loss of ultrafiltration capacity ensued which was fully rescued by paricalcitol treatment. Furthermore, PD-induced ECM thickening was significantly reduced and omental PD-induced angiogenesis was less pronounced upon paricalcitol treatment. No effect of paricalcitol treatment on total amount of peritoneal cells, peritoneal leukocyte composition, and epithelial to mesenchymal transition (EMT) was observed. Our data indicates that oral VDRA reduces tissue remodeling during chronic experimental PD and prevents loss of ultrafiltration capacity. Therefore, VDRA is potentially relevant in the prevention of treatment technique failure in PD patients.

## 1. Introduction

Peritoneal dialysis (PD) is a renal replacement therapy for patients with end-stage renal disease (ESRD). During long-term PD, morphological changes occur in the peritoneum including interstitial fibrosis leading to thickening of the membrane and neovascularization [1]. Together with the induction of inflammatory processes, this can lead to loss of peritoneal membrane function, technique failure, and premature discontinuation of PD therapy [2]. The mechanisms involved in these pathological changes are incompletely understood.

Vitamin D was originally identified as a key regulator for bone metabolism and calcium homeostasis. Novel insights revealed that its biological actions go beyond this and also include regulation of inflammation and angiogenesis, as well as cell growth and differentiation and apoptosis of many cell types [3, 4]. Chronic kidney disease (CKD) patients have low levels of active vitamin D as conversion of vitamin D_3_, 25-hydroxyvitamin D_3_ (25D), into the bioactive form, 1,25-dihydroxyvitamin D_3_ (1,25D), occurs mainly in the kidney [5].

Based on these previous findings we hypothesized that active vitamin D can attenuate or prevent the changes observed after long-term PD. To address this question we applied an experimental model of peritoneal dialysis.

## 2. Methods

### 2.1. Animals and Experimental Design

**Figure 1:**
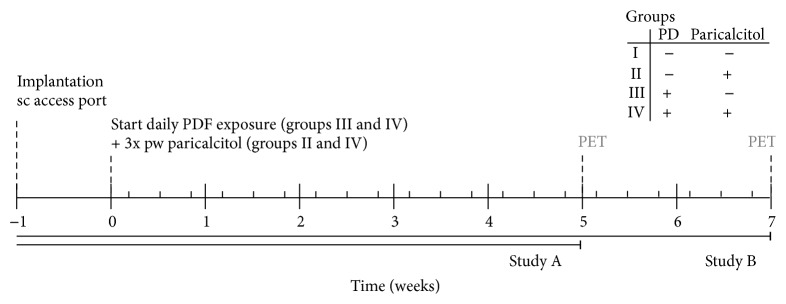
Time schedule animal experiments.

Male Wistar rats (Harlan CPB, Horst, Netherlands) weighing 280–330 grams were used in all experiments. After arrival, the rats were allowed one week of acclimatization. Animals were maintained under conventional laboratory conditions and were given food and water* ad libitum*. The experimental design (Figure 1) was approved by the Animal Care Committee of the VU University Medical Center, Amsterdam.

Animals were randomly assigned to one of four groups and treated for five weeks: I: controls receiving 3 times weekly sugar water (orally) as vehicle control (*n* = 9), II: control rats receiving 3 times weekly orally paricalcitol, dissolved in sugar water (40 ng/kg rat, Zemplar, kindly provided by AbbVie, Chicago, USA; *n* = 9), III: animals receiving daily instillation of 10 mL conventional PDF and 3 times per week sugar water according to the same regime as group I (Dianeal, 3.86% glucose, pH5.2, Baxter R&D, Utrecht, Netherlands; *n* = 13), and IV: rats receiving daily instillation of 10 mL conventional PDF and paricalcitol treatment according to the same regime as group II (*n* = 13). PDF was instilled via a subcutaneously implanted access port as described previously [6]. Oral administration of paricalcitol was achieved in the following way: the rats were taught to drink sugar water via a syringe when offered. In this way we could limit the discomfort for the animals caused by oral gavage. Since multiple animals were housed per cage dissolving paricalcitol in drinking water would lead to unknown amounts of paricalcitol administered per animal.

To corroborate our results, additional animals were also treated for seven weeks as described above (I: *n* = 8; II: *n* = 8; III: *n* = 15; IV: *n* = 15, resp.) with a small dose adjustment of the oral treatment of paricalcitol to 30 ng/kg rat paricalcitol, three times weekly, in order to maintain a stable cumulative dose of paricalcitol among treated groups.

### 2.2. Readouts

Table 1 summarizes the analysis performed after five or seven weeks of treatment.

#### 2.2.1. Analysis of Effluent

At the end of the experiment, a 90-minute peritoneal equilibrium test (PET) was performed under fentanyl citrate-fluanisone (0.05 mL/100-gram bodyweight; VetaPharma, Leeds, UK) and midazolam (0.08 mL/100-gram bodyweight; Actavis B.V., Baarn, Netherlands) anesthesia. 30 mL conventional PDF was instilled into the peritoneal cavity via a direct intraperitoneal catheter (Venflon Pro, BD Medical Systems, Franklin Lakes, NJ, USA). After drainage, the ultrafiltration capacity was calculated (effluent volume minus 30 mL) and the cell pellet was collected. Cell number and viability were determined in a hemocytometer by trypan blue exclusion. Cytocentrifuge preparations were stained with May-Grünwald-Giesma and cells were differentiated. Cell-free effluent was stored at −20°C or −80°C for determination of biomarkers for both five- and seven-week PETs.

In the animals treated for five weeks, glucose, creatinine, urea (GLUC3, CREA, and UREAL, resp., COBAS 8000, Roche Diagnostics, Basel, Switzerland), and sodium (ABL 800 FLEX, Radiometer, Zoetermeer, Netherlands) concentrations were analysed in serum samples, collected via a heart punction after sacrificing the animals by CO_2_/O_2_ induction, and in cell-free effluents and dialysis/serum (D/P) transport ratios were calculated. Based on a glucose determination of a pure Dianeal sample, and glucose detection in the cell-free effluent of the PETs, the percentage of glucose absorption was calculated. In the calculation a correction for the PET volume was included.

Hyaluronic acid (HA) was determined in the effluent using an ELISA-based assay according to Fosang et al. [7]. The concentrations TGF-*β* (TGF-*β*1, Promega GmbH, Manheim, Germany), VEGF (R&D systems, Abingdon, UK, or Milliplex MAP rat cytokine kits, Millipore, Billerica, MA, USA), and MCP-1 (Merck MILLIPORE, Darmstadt, Germany, or Milliplex MAP rat cytokine kits) were also analyzed in the PET effluents of both experiments. In addition, IL4, IL10, IL12p70, IL5, and GRO/KC (IL8 related protein in rodents) were analyzed only for the PET effluents collected after 7 weeks of PDF exposure using multiplex bead arrays (Milliplex MAP rat cytokine kit). Milliplex MAP is based on Luminex xMAP technology and used as recommended by the manufacturers.

#### 2.2.2. Morphological Analysis

Parietal peritoneum samples were taken at the contralateral side of the tip of the catheter. Cryostat sections were cut and stained with Van Gieson (Merck, Darmstadt, Germany) to quantify fibrosis. To determine the submesothelial thickness, images were analyzed by measuring, on average, 10 independent points per animal (Leica LAS AF version 2.6.0, Leica Microsystems, CMS GmbH, Mannheim, Germany). A part of the omentum, of both rats treated for five weeks and rats treated for seven weeks, and mesenteric tissue, only of rats treated for five weeks, was dissected and spread on a glass slide. To visualize vasculature and macrophages the tissues were stained with CD31 (PECAM; Serotec, Oxford, UK) and ED2 (Serotec, Oxford, UK). Images were analyzed by digital image analysis (AnalySIS Soft Imaging System, Olympus, Hamburg, Germany, or CellProfiler: image analysis software for identifying and quantifying cell phenotypes).

Liver imprints of the mesothelial monolayer were made, after seven weeks of treatment, by pressing 6% gelatine coated glass slides on the slightly dried liver after sacrificing, and stained for vimentin (Serotec, Oxford, UK), cytokeratin (DakoCytomation, Glostrup, Denmark), and DAPI (Invitrogen, Breda, Netherlands) to determine epithelial to mesenchymal transition [8]. On average seven images per rat were analyzed manually (Leica LAS AF version 2.6.0, Leica Microsystems, CMS GmbH, Mannheim, Germany), whereby increased vimentin expression and change in morphology from cobbled stone like cells towards spindle like cells were counted as cells that underwent EMT.

#### 2.2.3. Serum Analysis

Serum samples were analyzed for 25-hydroxyvitamin D3 by competitive binding protein assay (DiaSorin, Stillwater, Minnesota, USA), 1,25-dihydroxyvitamin D3 by radioimmunoassay after immunoextraction (IDS, Tyne and Wear, UK), PTH by ELISA (Scantibodies Laboratory, Santee, CA, USA), and calcium (Ca) and phosphate (P) by colorimetric assays (Roche Diagnostics, Mannheim, Germany). With exception of 25D, only measured in serum samples of animals treated for seven weeks, the analysis was performed after both five and seven weeks of treatment.

#### 2.2.4. *In Vitro* Macrophage Migration Assay

Macrophage migration was examined in Boyden transwell cell culture chambers using gelatine-treated polycarbonate membranes with 10 *μ*m pore size (Neuro Probe, Inc., Gaithersburg, MD, USA). Briefly, rat bone marrow cells were isolated and macrophages were allowed to adhere for 7 days in the presence of DMEM (Gibco, BRL, Gaithersburg, MD, USA) enriched with 15% v/v L-cell conditioned medium (LCM) and supplemented with 2% v/v penicillin-streptomycin-glutamine (PSG; Invitrogen, Breda, Netherlands) and 10% fetal calf serum (Biowest, Nuaillé, France). Other cells were washed away and macrophages were harvested by lidocaine treatment. Cells were resuspended in serum-free DMEM to a concentration of 2*∗*10^5^ cells/mL. Aliquots of 50 *μ*L were added to the upper chamber, while the lower chamber was filled with 25 *μ*L of DMEM containing MCP-1 (10 ng/mL), paricalcitol (1*∗*10^−6^ M), or a combination of both, with or without the addition of Dianeal (1 : 4 with DMEM). After 6 hours of incubation at 37°C, cells were removed from the upper chamber side of the membrane. The membrane was washed and stained with Coomassie. The cells on the bottom side of the filter were counted and expressed as percentage of migrated cells compared to control DMEM medium without chemoattractant. The experiment was performed in triplicate using different cell isolations.

### 2.3. Statistical Analysis

Data presented as median and interquartile range are analysed by using the Kruskal-Wallis test followed by Dunn's Multiple Comparison test to compare the following groups: I versus II, I versus III, I versus IV, and III versus IV. The detection limit was used for statistical analysis for VEGF, IL4, IL10, IL12p70, IL5, and GRO/KC levels in the effluent, when the concentrations were below detection limit.

## 3. Results

The well-being of all rats was monitored daily and no unexpected abnormalities were observed. Twelve of the total 56 animals exposed to PDF were taken out of the experiment due to abdominal fat or omental tissue wrapping around the tip of the catheter, which was consistent with previous experience [8]. All control animals (groups I and II), 25 out of 30 in group III and 19 out of 30 in group IV, remained for analysis after five and seven weeks of PDF exposure.

### 3.1. Vitamin D Mineral Homeostasis

1,25D levels showed a declining trend in paricalcitol treated animals after five weeks and a significant decrease after seven weeks of treatment compared to the control animals (Table 2). In addition, the same effect of paricalcitol on 25D levels was detected in the serum samples measured after seven weeks of treatment. Ca and P levels were not affected by paricalcitol treatment, except for animals in the control group receiving 40 ng/kg paricalcitol, which had significantly higher P level compared to the control animals.

### 3.2. Peritoneal Transport

**Figure 2:**
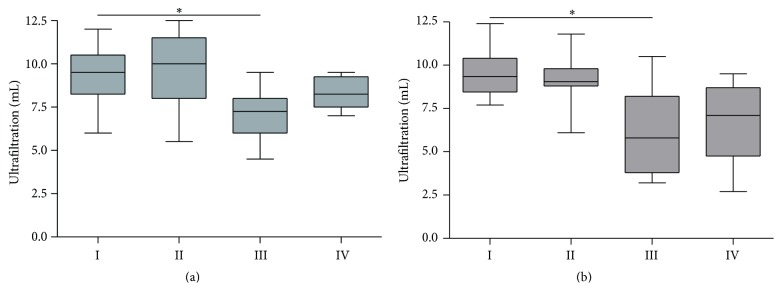
Net ultrafiltration after 90-minute PET. Net ultrafiltration after a 90-minute PET with 30 mL conventional PD fluid after five (a) and seven (b) weeks of treatment. All data presented as median and interquartiles. Whiskers indicate the extremes. ^*∗*^ *p* < 0.05 compared to group I.

The PET resulted in ~10 mL net ultrafiltration (UF) in control animals (groups I and II). Chronic PD treatment significantly reduced ultrafiltration capacity towards a median of 7.3 mL after five weeks and, even worse, 5.8 mL net UF after seven weeks (*p* = 0.05, group III versus group I; Figure 2). Paricalcitol treatment in the PDF-exposed group prevented these significant changes in UF (*p* > 0.05 versus group I) and resulted in a 10–15% increase in UF capacity compared to PDF exposure alone.

To further analyze the effect of PDF exposure and vitamin D receptor activation on peritoneal functional decline, transport parameters were measured in serum and PET effluents after five weeks of treatment (Table 3). Exposure to PDF changed D/P creatinine from 0.2 to 0.4 (*p* < 0.01 versus group I). Paricalcitol did not affect the D/P creatinine in both control and PD treated rats. Although compared to control levels (group I) D/P urea (0.5) and D/P sodium (0.8) were slightly higher in group III (0.6, *p* > 0.05, and 0.9, *p* < 0.05, resp.) and group IV (0.6, *p* < 0.01, and 0.8, *p* > 0.05, resp.), absolute changes in ratios were minimal. Glucose absorption increased, although not significantly, from 33.3% to 42.8% upon PDF exposure. Paricalcitol treatment lowered, but not significantly, glucose absorption in both non-PDF-exposed and PDF-exposed animals (33.3% in group I versus 22.3% in group II) and PDF-exposed animals (46.5% in group III versus 42.8% in group IV).

### 3.3. Cell Numbers and Macrophage Migration

**Figure 3:**
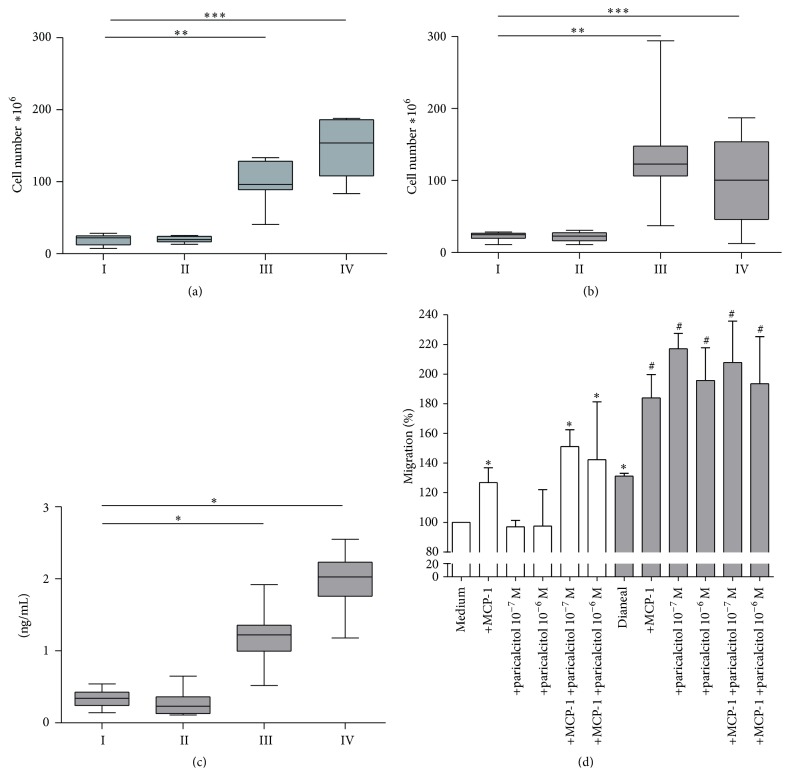
Cell numbers and macrophage migration. Total peritoneal cell number in effluent after 90-minute PET with 30 mL of conventional PD fluid after five (a) and seven (b) weeks of treatment; ^*∗∗*^ *p* < 0.01; ^*∗∗∗*^ *p* < 0.001. Effluent concentration of MCP-1 after five weeks of treatment (c); ^*∗*^ *p* < 0.05. Rat macrophage migration towards paricalcitol; MCP-1 in standard medium (white bars) or medium containing Dianeal (1 : 4) (grey bars; (d) ^*∗*^ *p* < 0.05 versus medium; ^#^ *p* < 0.05 versus Dianeal + medium).

Total cell numbers increased significantly upon PD treatment and were approximately five times higher compared to control animals (*p* < 0.01 and *p* < 0.001 for groups III and IV versus group I, resp.; Figures 3(a) and 3(b)). However, there was no significant difference between the paricalcitol and vehicle control treated groups. Cell differentiation of peritoneal cells in the effluents revealed a reduction of eosinophil and mast cell count and an increase in neutrophils after PD treatment. In all groups macrophages remained the dominant cell type (±80%; Table 4). Paricalcitol treatment did not induce significant differences in leukocyte composition in control or PD treated rats after five or seven weeks of PDF exposure.

### 3.4. Analysis of Peritoneal Effluents and* In Vitro* Macrophage Migration

MCP-1 was measured in the PET effluents and showed to be unaffected by paricalcitol treatment in the control situation (group II, Figure 3(c)). After five weeks of PD treatment, MCP-1 levels were ~5-fold increased in PD rats compared to control rats (*p* = 0.05). Paricalcitol treatment in combination with PD tended to increase MCP-1 levels even further, although not significantly, to a median of 2 ng/mL compared to 1.2 ng/mL in group III and 0.3 ng/mL in both control groups. No difference was found in MCP-1 levels between the different groups in the animals treated for seven weeks.

To examine the effect of paricalcitol and peritoneal dialysis fluid on macrophage migration,* in vitro* migration assays with primary rat macrophages were performed. As expected, MCP-1 induced macrophage migration. Paricalcitol did not change migration under these (control) conditions (Figure 3(d), white bars). However, when Dianeal was added to the culture medium (1 : 4), which is known to result in macrophage activation [9], paricalcitol enhanced migration, similar to the levels of MCP-1 induced migration (*p* < 0.05) (Figure 3(d), grey bars). Simultaneous addition of paricalcitol and MCP-1 did not further increase macrophage migration when Dianeal was present.

**Figure 4:**
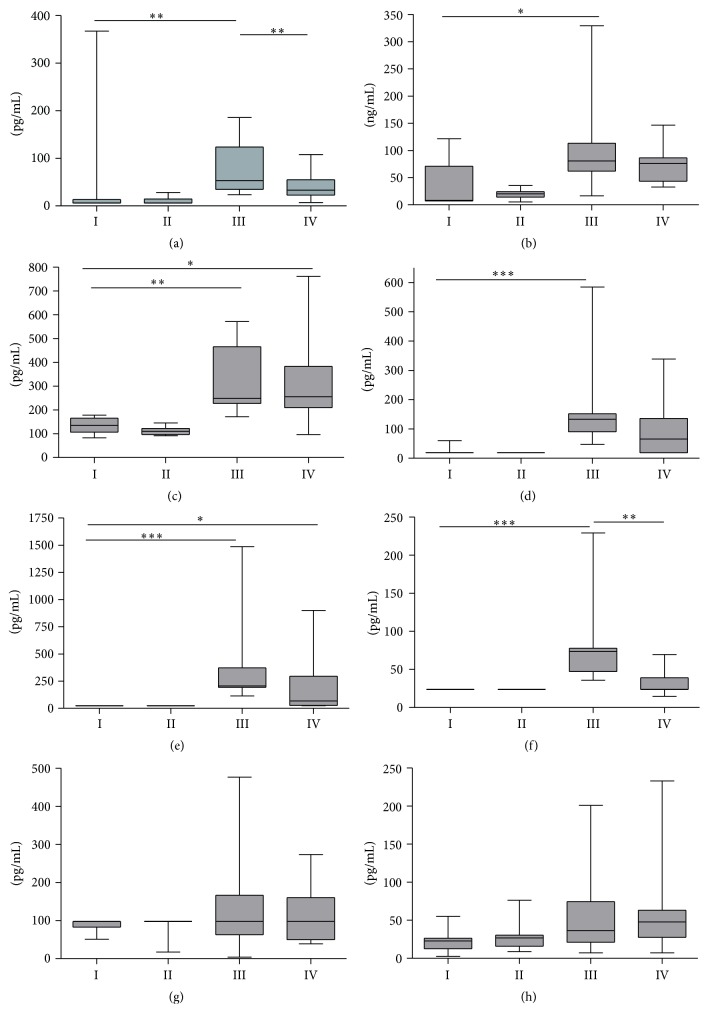
Peritoneal effluent concentrations. VEGF (a), HA (b), TGF-beta (c), IL4 (d), IL12p70 (e), IL5 (f), IL10 (g), and GRO/KC (h) levels in the peritoneal effluent after seven weeks of treatment. Data presented as median and interquartiles. Whiskers indicate the extremes. ^*∗*^ *p* < 0.05; ^*∗∗*^ *p* < 0.01; ^*∗∗∗*^ *p* < 0.001.

VEGF levels in peritoneal effluents were below detection limits in most control animals but were statistically significantly higher in the PDF-exposed groups. Seven weeks of paricalcitol treatment reduced VEGF levels to 40 pg/mL (median) compared to 72 pg/mL in the PDF-exposed group (*p* = 0.01; Figure 4(a)). However, after five weeks, no effect of paricalcitol treatment on effluent VEGF concentration was found (data not shown).

As shown previously [8, 10], PD treatment induced a significant increase in HA production, indicating an inflammatory state in the peritoneum (*p* = 0.001) (Figure 4(b)). Paricalcitol treatment mitigated the increase in HA concentrations in the rats exposed to PDF after five and seven weeks of treatment.

In PD treated animals, TGF-*β* concentrations were on average 2.7 times higher compared to control animals (*p* = 0.05) after five (data not shown) or seven (Figure 4(c)) weeks of treatment. Paricalcitol treatment did not affect the TGF-*β* levels in the effluent.

IL4 levels were below detection limit in almost all animals in the control groups (18 pg/mL; groups I and II). IL4 levels were significantly increased in group III compared to group I (*p* = 0.001) with an average of 186 pg/mL. Although there was no significant difference between groups III and IV, IL4 levels were less pronounced upon paricalcitol treatment with an average concentration of 92 pg/mL (Figure 4(d)).

IL12p70 concentrations in the effluent significantly increased upon PD treatment in groups III and IV compared to group I, in which all levels were found to be below detection limit (24 pg/mL); *p* = 0.001 and *p* = 0.05, respectively. A nonsignificant trend was observed whereby, upon PDF exposure, paricalcitol mitigated the rise of IL12p70 with a median concentration of 68 pg/mL compared to 207 pg/mL in group III (Figure 4(e)).

Also IL5 concentrations were below detection limit (24 pg/mL) in all control animals (groups I and II). PDF exposure alone led to an increase of IL5 concentrations in the effluent (74 pg/mL), whereas paricalcitol treatment completely prevented this significant elevation (24 pg/mL; *p* = 0.01 versus group III; Figure 4(f)).

Even though a few animals in the PDF-exposed groups had IL10 levels above the detection limit, the medians of all groups were 98 pg/mL IL10, so no difference was determined (Figure 4(g)). GRO/KC concentrations were not significantly increased upon PDF exposure and no effect of paricalcitol treatments was observed. Median GRO/KC levels were 23, 27, 37, and 48 pg/mL for groups I, II, III, and IV, respectively (Figure 4(h)).

### 3.5. Peritoneal Tissue Remodeling

**Figure 5:**
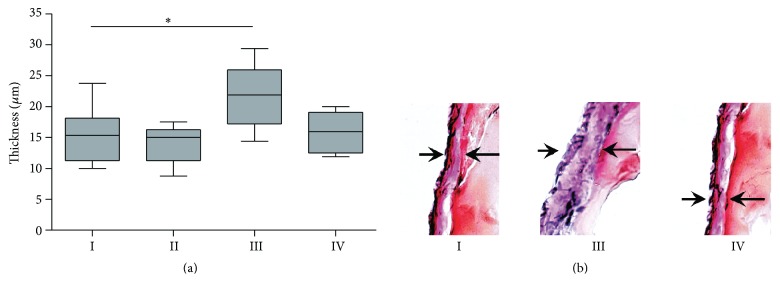
Extra cellular matrix thickness. ECM thickness of parietal peritoneum (a) and representative examples of peritoneal sections of groups I, III, and IV, respectively, after 5 weeks of treatment. All data presented as median and interquartiles. Whiskers indicate the extremes. ^*∗*^ *p* < 0.05.

Histological analysis showed that five-week PDF exposure resulted in increased submesothelial matrix thickness (median 22 *μ*m) compared to control rats (median 15 *μ*m; *p* = 0.05 versus group I; Figure 5). Additional paricalcitol treatment prevented thickening of the parietal mesothelial matrix layer (16 *μ*m; *p* > 0.05 versus group I). After seven weeks of PDF exposure no difference in submesothelial matrix thickness was found between all groups, thus also not between PDF and non-PDF treated groups (data not shown).

**Figure 6:**
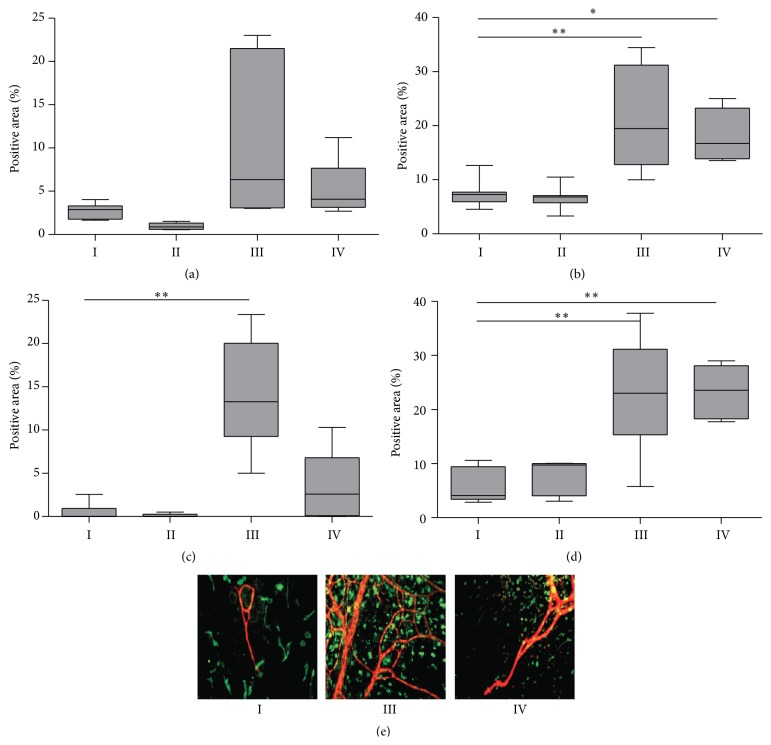
Angiogenesis and macrophage accumulation in visceral peritoneum. Macrophage accumulation (Ed2 positive staining) in omentum (a) and mesentery (b) and angiogenesis (CD31 positive staining) in omentum (c) and mesentery (d) after 5 weeks of PDF exposure and paricalcitol treatment. Representative examples of the omentum with ED2 in green and CD31 in red of control rat (group I), PDF-exposed rat (group III), and PDF-exposed rat treated with paricalcitol (group IV) (e). All data presented as median and interquartiles. Whiskers indicate the extremes. ^*∗*^ *p* < 0.05; ^*∗∗*^ *p* < 0.01.

Chronic PD treatment resulted in increased recruitment of activated M2 tissue macrophages and in new vessel formation in omentum and mesentery, determined by, respectively, ED2 and CD31 staining (Figure 6). In the mesentery, paricalcitol treatment could not prevent PD-induced macrophage accumulation or angiogenesis (Figures 6(b) and 6(d)). However, a declining trend in median omental ED2 positive macrophage accumulation was observed during paricalcitol treatment for both the control (group II 1% versus group I 3% positive area) and the PDF-exposed groups (group IV 5% versus group III 10% positive area; Figures 6(c) and 6(e)). Moreover, PD-induced angiogenesis in the omentum (14% positive area; *p* = 0.01 compared to group I) was less pronounced for paricalcitol treated animals (4% positive area; *p* > 0.05 compared to group I) after five weeks of treatment (Figures 6(d) and 6(e)). Although no significant differences in omental CD31 positive area were observed after seven weeks between groups I and III, a declining trend in the CD31 positive area upon paricalcitol treatment was observed (33% versus 14% and 32% versus 27% for groups I versus II and III versus IV, resp.; data not shown).

### 3.6. Liver Imprints

**Figure 7:**
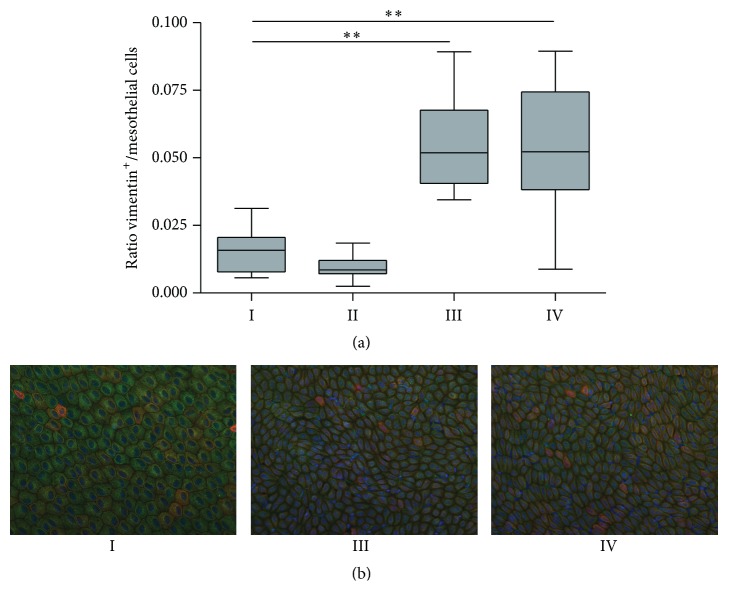
Epithelial to mesenchymal transition on liver imprints. Ratio vimentin positive/mesothelial cells on liver imprints (a). Representative examples of liver imprints with nuclei in blue, vimentin in green, and cytokeratin in red of control rat (group I), PDF-exposed rat (group III), and PDF-exposed rat treated with paricalcitol (group IV) (b). All data presented as median and interquartiles. Whiskers indicate the extremes. ^*∗∗*^ *p* < 0.01.

Liver imprints were taken after seven weeks of PDF exposure. Mesothelial cell density and the number of vimentin positive-cytokeratin negative cells increased in the PDF-exposed animals (Figure 7(a); groups III and IV compared to group I) indicating mesothelial cell regeneration and epithelial to mesenchymal transition. In addition, the cobblestone appearance of the mesothelial cells (Figure 7(b), group I) is partly lost in groups III and IV, in which the cells are more stretched. The ratio vimentin positive-cytokeratin negative cells/mesothelial cells approximately doubled in the PDF-exposed groups (III and IV) compared to the control (group I). Paricalcitol treatment did not influence this process (Figure 7(a)).

## 4. Discussion

In the present study, the role of VDR activation in peritoneal remodeling in a chronic rat PD model was investigated while validating our model by comparing the control and PDF exposure only groups as well (group I versus group III). In the animals exposed to PDF, compared to the control situation, we observed worsening of ultrafiltration capacity, elevation in inflammation markers, partly increased vascular surface area, and a higher number of cells undergoing epithelial to mesenchymal transition. This is in line with previous observations [8, 10]. Paricalcitol treatment influenced several of the examined parameters. Loss of ultrafiltration capacity, increase in ECM thickness, angiogenesis, and IL5 levels due to PDF exposure were significantly attenuated by paricalcitol treatment. In addition, a trend towards decreased glucose absorption, less ED2 positive macrophage accumulation in the omentum and mesentery, and lower HA, VEGF, IL12p70, and IL4 levels was observed upon paricalcitol treatment in PDF-exposed animals. However, not all factors involved in peritoneal remodeling upon PD were affected by paricalcitol treatment, such as total cell number and epithelial to mesenchymal transition.

Importantly, paricalcitol treatment decreased endogenous 1,25D levels, which has also been found by others in both animal and human studies [11, 12] and is the consequence of upregulation of the catabolic enzyme 25(OH)D-24-hydroxylase. This indicates successfulness of applying oral paricalcitol treatment in our rat model.

The main finding of this paper is the demonstration that paricalcitol can attenuate the loss in ultrafiltration capacity upon PDF exposure. Driving forces for ultrafiltration in peritoneal dialysis are the maintenance of an osmotic gradient and the existence of low barrier resistance for water transport. Glucose absorption showed a slight, although not significant, increase after five weeks of PDF exposure, indicating a small loss of osmotic gradient. In the control situation, and to lesser extent in the PDF-exposed group, addition of paricalcitol resulted in a trend towards positively affecting the osmotic gradient. Despite the fact that the trend was small and not significant, this could have contributed to the partial preservation of the ultrafiltration capacity in animals receiving oral paricalcitol in a PD environment. Consistent with this is the observed attenuated neovascularization by paricalcitol. Neovascularization results in increased perfusion of the peritoneal membrane, which is considered to be one mechanism leading to enhanced dissipation of the osmotic gradient, by early enhanced glucose uptake from the peritoneal dialysis fluid.

In line with the observed partial prevention of ultrafiltration failure by paricalcitol treatment, we showed prevention of increasing ECM thickness, which may indicate a lower barrier resistance for water transport, upon PDF exposure after five weeks of treatment with paricalcitol. This latter finding is in accordance with the compelling evidence that vitamin D treatment can reduce fibrosis [13, 14]. Possible explanations for reduced fibrosis after paricalcitol treatment may be found in proteins involved in the thickening of the ECM-layer such as collagen type-1 and the renin-angiotensin-aldosterone (RAAS) system, which are downregulated upon VDR activation [15, 16].

The immunomodulatory effects of paricalcitol could have beneficially contributed to the development of ECM thickness. In our experiments paricalcitol modulated the concentrations of IL5 and IL4 and possibly also affected HA and VEGF, factors derived from cells of the immune system and/or mesothelial cells. Although we cannot prove the direct effect of these cytokines on peritoneal membrane remodeling, our data are in line with a recent study showing the importance of cytokines by correlating reduction in IL-17 and activation of regulatory T-cells with reduced fibrosis [17].

In our experiments paricalcitol treatment led to a ~50% decreased population of ED2 positive “M2” macrophages in the omentum in both control and PDF-exposed rats, which might have led to lesser thickening of the submesothelial matrix we observed after five weeks of treatment. In other studies, it has also been shown that macrophages play an important role in fibrosis, whereby M2 macrophages correlate with fibrosis in sclerotic skin and pulmonary and kidney fibrosis [18–21]. Moreover, there is compelling evidence that M2 macrophages are involved in peritoneal fibrosis in PD [22, 23]. In addition, paricalcitol treatment mitigated IL12p70 concentrations, whose production is related to M1 macrophages [24], upon PDF exposure.

Vitamin D receptor activation has acknowledged antiangiogenic properties [25]. In this study it is also shown that paricalcitol tended to reduce angiogenesis, especially in the omentum. This observation is in line with previous* in vitro *studies. These studies demonstrate reduced proliferation of human umbilical vein endothelial cells upon paricalcitol treatment [26]. In addition, in a mouse model for PD, it has been shown that paricalcitol can prevent angiogenesis [17]. Several humoral factors can be involved in this reduced angiogenesis following paricalcitol. IL12p70, however, which is described to have antiangiogenic properties, declined in the paricalcitol group. This suggests that the effects of paricalcitol are not mediated by this factor. VEGF is another prominent proangiogenic factor [27]. Here, we show indeed that the PDF exposure-induced increase in VEGF, after seven weeks of treatment, is attenuated by paricalcitol treatment. Finally, MCP-1, which has been shown also to have proangiogenic capacities [28], was not reduced by paricalcitol.

In line with previous studies, we found an increase in peritoneal cells observed after PD treatment. The higher cell numbers could be due to the enhanced levels of chemoattractants such as MCP-1 and IL5 [10, 29]. Although paricalcitol did not influence total cell numbers, as described above, it tended to influence the type of cells including the decrease of ED2 positive cells.

The ratio vimentin positive/mesothelial cells, as indicator of EMT, increased upon PDF exposure compared to the control situation, which makes our model suitable to study the effects of paricalcitol on EMT. However, contrary to other studies, we were not able to find a decrease in EMT which might be due to the different models or concentrations of paricalcitol used [30, 31].

To summarize, although we did not observe an effect of paricalcitol on EMT, parts of the effects of paricalcitol are found to be consistent. Paricalcitol partly preserved the ultrafiltration capacity upon PDF exposure. We observed partial prevention of angiogenesis, and thus a smaller vascular surface area, which could contribute to the observed trend in preservation of the glucose driven osmotic gradient. In addition, prevention of increase in ECM thickness was found, which indicates less resistance of the peritoneal membrane and thus a better ultrafiltration capacity.

Our study has several limitations. Firstly, differences between the control and PDF-exposed groups, such as ultrafiltration capacity and ECM thickness, were less pronounced after seven weeks of PDF exposure. This was likely caused by the apparent less mesothelial toxic effects in this particular control group of animals. We know from earlier experiments that differences in separate experiments may occur. Besides sampling error and inequal distribution of toxicity could be of importance too. Therefore, we could have missed potential protective effects of paricalcitol treatment in this group due to a lack of pathological changes in the PD control animals. However, we did use a wide range of additional parameters in which morphological, functional, and biochemical components were included and did observe all well-described changes in the PD treated groups for five weeks.

A second limitation is that we did not use uremic model and vitamin D deficient model. However, we hypothesize that under those conditions the effect of VDR activation is likely even more pronounced.

Taken together, we have shown that VDR activation can partly restore ultrafiltration failure due to limiting ECM thickening and angiogenesis even in a calcitriol sufficient environment. Future studies should be carried out to address the clinical benefit of improved PD efficacy on ultrafiltration in particular.

## Figures and Tables

**Table 1:** Analyses performed after 5 and/or 7 weeks of treatment.

Analysis	Time period treatment	
	5 weeks	7 weeks
Effluent		
Ultrafiltration	+	+
Transport parameters	+	−
Cell count	+	+
Cell differentiation	+	+
Hyaluronic acid	+	+
TGF-beta	+	+
VEGF	+	+
MCP-1	+	+
IL4	−	+
IL10	−	+
IL12p70	−	+
IL5	−	+
GRO/KC	−	+
Morphological		
ECM thickness	+	+
Liver imprints	−	+
Omentum		
Vasculature	+	+
Macrophages	+	+
Mesentery		
Vasculature	+	−
Macrophages	+	−
Serum		
Transport parameters	+	−
25D	−	+
1,25D	+	+
Ca	+	+
P	+	+

+ indicates the analysis has been performed; − indicates the analysis has not been performed.

**Table 2:** Effect of paricalcitol on mineral homeostasis.

Treatment	Five weeks				Seven weeks			
Group	I	II	III	IV	I	II	III	IV

1.25 vitamin D3 (pmol/L)	436.0	342.0	564.0	310.5	255.5	33.5^a^	384	42^b^
IQR	[352.8–519.3]	[256.0–427.0]	[427.5–673.5]	[288.0–365.3]	[225–279]	[30.8–36]	[330.8–456.8]	[39–45]
25 vitamin D3	No data	No data	No data	No data	63	39^a^	75.5	52^b^
IQR					[59.5–70.8]	[37.8–40.5]	[69.3–79.8]	[51–54]
Phosphate (mmol/L)	3.4	4.6^a,b^	3.3^a^	3.2	3.0	3.4	3.6	4.1
IQR	[2.7–4.1]	[4.0–4.7]	[3.2–3.4]	[2.8–3.6]	[3.0–3.2]	[3.1–3.5]	[3.6–4.5]	[3.6–5.4]
Calcium (mmol/L)	3.6	3.6	3.5	3.4	3.2	3.1	3.4	3.4
IQR	[3.6–3.8]	[3.4–3.8]	[3.2–3.5]	[3.3–3.5]	[3.1–3.3]	[3.2–3.0]	[3.3–3.6]	[3.4–3.2]

All data presented as median and interquartiles.

^a^ *p* < 0.05 compared to group I; ^b^ *p* < 0.05 compared to group III.

**Table 3:** Peritoneal transport parameters determined by PET effluent D/serum P after 5 weeks of treatment.

Group	I	II	III	IV
Glucose absorption (%)	33.3	22.3	46.5	42.8
	[28.5–38.5]	[21.5–28.2]	[35.6–53.7]	[36.9–48.8]
D/S creatinine	0.2	0.2	0.4^b^	0.4^b^
	[0.1–0.2]	[0.2–0.3]	[0.3–0.5]	[0.3–0.5]
D/S urea	0.5	0.5	0.6	0.6^b^
	[0.4–0.5]	[0.4–0.5]	[0.5–0.7]	[0.6–0.7]
D/S sodium	0.8	0.8	0.9^a^	0.8
	[0.6–0.8]	[0.7–0.8]	[0.8–0.9]	[0.8–0.9]

All data presented as median and interquartiles.

^a^ *p* < 0.05 compared to group I; ^b^ *p* < 0.01 compared to group I.

**Table 4:** Composition of peritoneal leukocytes.

Treatment	Five weeks				Seven weeks			
Group	I	II	III	IV	I	II	III	IV

Cell number ×10^6^	22.1	19.5	99.8^b^	153.9^b^	24.95	22.9	122.75	92.25
IQR	[12.5–24.8]	[16.2–24.2]	[89.0–130.1]	[108.1–186.1]	[20.3–26.7]	[17.3–26.3]	[109.4–144.9]	[46.9–153.4]
Macrophages (%)	77.0	80.0	84.7	78.9	89	88.5	88	86.5
IQR	[74.0–81.0]	[76.6–82.1]	[72.3–90.4]	[68.8–87.7]	[85.8–90.3]	[87.5–90.3]	[85.8–92.5]	[79.5–91.8]
Lymphocytes (%)	0.0^a^	0.0	0.5^c^	0.4	0	0	1	3
IQR	[0.0–0.0]	[0.0–0.0]	[0.3–1.3]	[0.0–0.9]	[0–1.25]	[0–0.3]	[0.5–1.8]	[0.8–4.5]
Neutrophils (%)	0.0	0.0	7.25^c^	14.1^c^	0.5	0	4	2
IQR	[0.0–0.0]	[0.0–0.0]	[2.5–25.8]	[10.6–20.2]	[0–1]	[0–0]	[0–8.8]	[0–9]
Eosinophils (%)	11.3	13.8	2.3^a^	1.75	10.5	11	3^b^	5.5^a^
IQR	[9.0–13.9]	[11.3–14.6]	[1.5–5.4]	[0.6–11.9]	[9.8–11.3]	[9.8–11.8]	[1.8–5.3]	[2–6.5]
Mast cells (%)	9.8	7.3	0.0^c^	0.0^c^	0	0	0	0
IQR	[8.8–13.6]	[4.5–10.4]	[0.0–0.3]	[0.0–0.2]	[0–0]	[0–0]	[0–0]	[0–0]

All data presented as median and interquartiles.

^a^ *p* < 0.05 compared to C group; ^b^ *p* < 0.01 compared to C group; ^c^ *p* < 0.001 compared to C group.
